# The Anti-Inflammatory and Antimicrobial Potential of Selected Ethnomedicinal Plants from Sri Lanka

**DOI:** 10.3390/molecules25081894

**Published:** 2020-04-20

**Authors:** Mayuri Napagoda, Jana Gerstmeier, Hannah Butschek, Sudhara De Soyza, Simona Pace, Sybille Lorenz, Mallique Qader, Sanjeeva Witharana, Ajith Nagahawatte, Gaya Wijayaratne, Aleš Svatoš, Lalith Jayasinghe, Andreas Koeberle, Oliver Werz

**Affiliations:** 1 Department of Biochemistry, Faculty of Medicine, University of Ruhuna, Galle 80000, Sri Lanka; sudhara.gamini@yahoo.com; 2 Department of Pharmaceutical/Medicinal Chemistry, Institute of Pharmacy, Friedrich-Schiller-University Jena, 07743 Jena, Germany; jana.gerstmeier@uni-jena.de (J.G.); hannah_butschek@gmx.de (H.B.); simona.pace@uni-jena.de (S.P.); andreas.koeberle@uni-jena.de (A.K.); 3 Research Group Mass Spectrometry and Proteomics, Max Planck Institute for Chemical Ecology, 07745 Jena, Germany; lorenz@ice.mpg.de (S.L.); svatos@ice.mpg.de (A.S.); 4 National Institute of Fundamental Studies, Kandy 20000, Sri Lanka; mallique.qader@gmail.com (M.Q.); ulbj2003@yahoo.com (L.J.); 5 Faculty of Engineering, Higher Colleges of Technology, PO Box 4793 Abu Dhabi, UAE; switharana@hotmail.com; 6 Department of Microbiology, Faculty of Medicine, University of Ruhuna, Galle 80000, Sri Lanka; ajithnagahawatte@yahoo.co.uk (A.N.); gayabw@yahoo.co.uk (G.W.)

**Keywords:** 5-lipoxygenase, microsomal prostaglandin E_2_ synthase, nitric oxide, medicinal plants, antimicrobial, disinfectant

## Abstract

Traditional folk medicine in Sri Lanka is mostly based on plants and plant-derived products, however, many of these medicinal plant species are scientifically unexplored. Here, we evaluated the anti-inflammatory and antimicrobial potency of 28 different extracts prepared from seven popular medicinal plant species employed in Sri Lanka. The extracts were subjected to cell-based and cell-free assays of 5-lipoxygenase (5-LO), microsomal prostaglandin E_2_ synthase (mPGES)-1, and nitric oxide (NO) scavenging activity. Moreover, antibacterial and disinfectant activities were assessed. Characterization of secondary metabolites was achieved by gas chromatography coupled to mass spectrometric (GC-MS) analysis. *n*-Hexane- and dichloromethane-based extracts of *Garcinia cambogia* efficiently suppressed 5-LO activity in human neutrophils (IC_50_ = 0.92 and 1.39 µg/mL), and potently inhibited isolated human 5-LO (IC_50_ = 0.15 and 0.16 µg/mL) and mPGES-1 (IC_50_ = 0.29 and 0.49 µg/mL). Lipophilic extracts of *Pothos scandens* displayed potent inhibition of mPGES-1 only. A methanolic extract of *Ophiorrhiza mungos* caused significant NO scavenging activity. The lipophilic extracts of *G. cambogia* exhibited prominent antibacterial and disinfectant activities, and GC-MS analysis revealed the presence of fatty acids, sesquiterpenes and other types of secondary metabolites. Together, our results suggest the prospective utilization of *G.* *cambogia* as disinfective agent with potent anti-inflammatory properties.

## 1. Introduction

Sri Lanka, an island with a varied climate and topography of 65,610 km^2^ extension located in the Indian Ocean, displays a rich biodiversity distributed within a wide range of ecosystems. Although the country is relatively small in size, it has the highest plant diversity per unit area among Asian countries, and the vegetation supports over 3700 flowering plant species. It is estimated that more than 1400 plants are employed in the indigenous medicine in Sri Lanka [1]. 

The use of medicinal plants for the treatment of inflammatory disorders is a common practice in local communities in Sri Lanka, as evidenced by ethnobotanical surveys carried out in two administrative regions in the country [2,3]. Similarly, a number of plant species have been documented as antimicrobial remedies as well as disinfectants or antiseptics [4]. However, only a limited number of in-depth scientific studies have been conducted so far on the phytochemistry and bioactivities of these folklore plants.

Scientific studies on the anti-inflammatory activities of extracts from Sri Lankan plants were mainly based on classical in vivo models including rat paw edema [5,6,7]. Although such in vivo studies allow to judge the anti-inflammatory efficacy of the plant extracts, for example by showing reduction of swelling or pro-inflammatory cell infiltration as read-outs, they are however, inappropriate to reveal the underlying mechanisms of action. Moreover, the recent trend in the field of inflammation research is to search for alternative therapeutic agents from natural sources that are devoid of the adverse effects characteristic for conventional steroids or nonsteroidal anti-inflammatory drugs (NSAIDs). In this context, phytochemical studies and biochemical investigations on the mode of action of traditional complementary remedies are of utmost importance. For example, the identification of secondary metabolites that can interfere with multiple enzymes in eicosanoid biosynthesis is one of the current research foci to combat different inflammation-related diseases like rheumatoid arthritis, osteoarthritis, gout, inflammatory bowel disease, asthma, cancer, cardiovascular disease, and central nervous system-related disorders such as depression and Parkinson’s disease [8]. Since the pro-inflammatory eicosanoids produced by 5-lipoxygenase (5-LO; i.e., leukotrienes) and microsomal prostaglandin E_2_ synthase (mPGES)-1 (i.e., PGE_2_) are causative for the underlying pathology of the abovementioned disease conditions, inhibition of 5-LO and mPGES-1 is considered as a promising treatment approach [8,9]. 

In continuation of previous studies evaluating the 5-LO- and/or mPGES-1-inhibitory action of medicinal plants from Sri Lanka, that is, *Leucas zeylanica* [10], *Plectranthus zeylanicus* [11] and *Munronia pinnata* [12], the present in vitro investigations were undertaken to explain the anti-inflammatory properties of extracts prepared from popular medicinal plants in Sri Lanka that are extensively used in folk medicine to treat various inflammatory conditions (Table A1). The plants investigated were *Argyreia populifolia* Choisy (Family: Convolvulaceae), *Garcinia cambogia* (Gaertn.) Desr. (Family: Clusiaceae), *Hibiscus furcatus* Willd (Family: Malvaceae), *Mollugo cerviana* (L.) Ser. (Family: Molluginaceae), *Nyctanthes arbor-tristis* (L.) Gaertn. (Family: Oleaceae), *Ophiorrhiza mungos* L. (Family: Rubiaceae) and *Pothos scandens* L. (Family: Araceae). In addition to 5-LO and mPGES-1 inhibition, the nitric oxide (NO) scavenging potential was evaluated in this study. The immunoregulatory function of NO and its contribution to the inflammatory response is well established [13]. Thus, the biosynthesis of NO is increased in animal models of rheumatoid arthritis where NO synthase (NOS) inhibitors displayed strong protective, anti-inflammatory activities [14]. 

Apart from their application as anti-inflammatory medications, the above plant species have also been employed as antiseptics and as antimicrobial remedies in indigenous medicine [4]. In particular, remarkable antibacterial and antioxidant activities in different *Garcinia* species was reported [15,16,17]. Therefore, the antibacterial activities of the plant extracts were evaluated against Gram-positive and Gram-negative bacteria and the disinfectant capacity of the most potent extracts were also studied to determine the suitability of the plants for the potential use as herbal disinfective agents.

## 2. Results

### 2.1. Inhibition of 5-LO by the Plant Extracts

As a commonly applied strategy to elucidate the bioactivities of plants, which we exploited before to study Sri Lankan plants for anti-inflammatory potential [10,11,12], we successively extracted the plant material with solvents of increasing polarity, that is, starting with *n*-hexane, followed by dichloromethane (DCM), ethyl acetate, and then methanol. The inhibition of 5-LO product biosynthesis by the respective extracts was studied in vitro using isolated human neutrophils that were stimulated with the Ca^2+^-ionophore A23187 plus 20 µM arachidonic acid (cell-based assay) as well as using isolated human recombinant 5-LO as enzyme source and 20 µM arachidonic acid as substrate (cell-free assay). All extracts were initially screened at a concentration of 10 µg/mL, and for those extracts that caused >50% inhibition of 5-LO activity, the IC_50_ values were determined by conducting more detailed concentration-response experiments. As can be seen from Table 1**,** extracts from *G. cambogia, H. furcatus, M. cerviana* and *O. mungos* caused inhibition of 5-LO with strong potencies.

**Figure 1:**
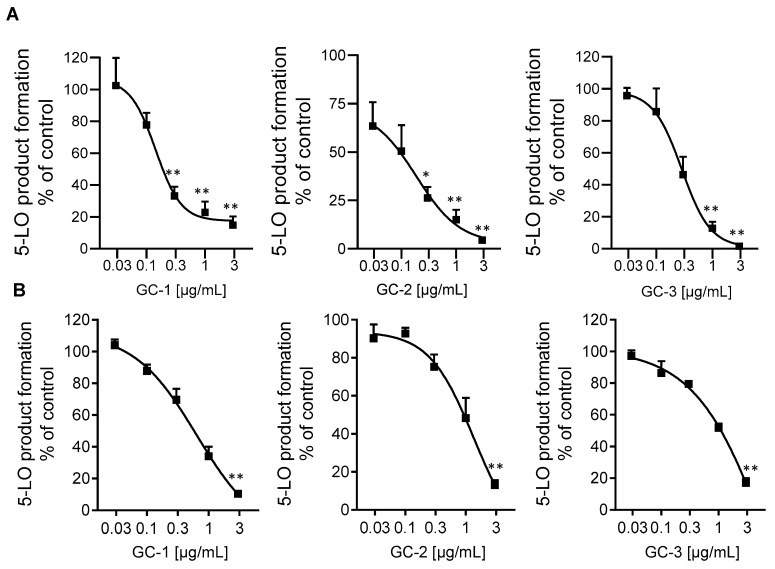
Inhibition of 5-LO in cell-free assays (**A**) and in intact neutrophils (**B**) by extracts of *n*-hexane (GC-1), DCM (GC-2) and ethyl acetate (GC-3) of *G. cambogia*. Data are given as means +/− S.E.M, *n* = 3. * *p* < 0.05; ** *p* < 0.01.

Most potent inhibition of 5-LO was observed with extracts of *G. cambogia* using *n*-hexane, DCM and ethyl acetate as solvent, yielding IC_50_ values of 0.15, 0.16, and 0.24 µg/mL, respectively, in the cell-free assay (Table 1, Figure 1A), being comparable to the potency of the approved anti-asthmatic drug zileuton [18] which was used as synthetic reference inhibitor with IC_50_ = 0.11 µg/mL, Table 1). These extracts caused also potent suppression of 5-LO activity in intact neutrophils (IC_50_ = 0.92, 1.39, and 1.55 µg/mL, respectively), with the *n*-hexane extract being most effective (Table 1, Figure 1B). The IC_50_ value of zileuton was determined as 0.13 μg/mL, corresponding to 0.55 μM (Table 1). In addition, the ethyl acetate extract of *H. furcatus* (IC_50_ = 1.0 µg/mL), the *n*-hexane extract of *M. cerviana* (IC_50_ = 5.5 µg/mL) and the methanol extract of *O. mungos* (IC_50_ = 1.6 µg/mL) were also promising candidates in the search for natural 5-LO inhibitors (Table 1), reflected by relatively low IC_50_ values compared to those of other plant extracts reported in literature [19].

### 2.2. Inhibition of mPGES-1

**Figure 2:**
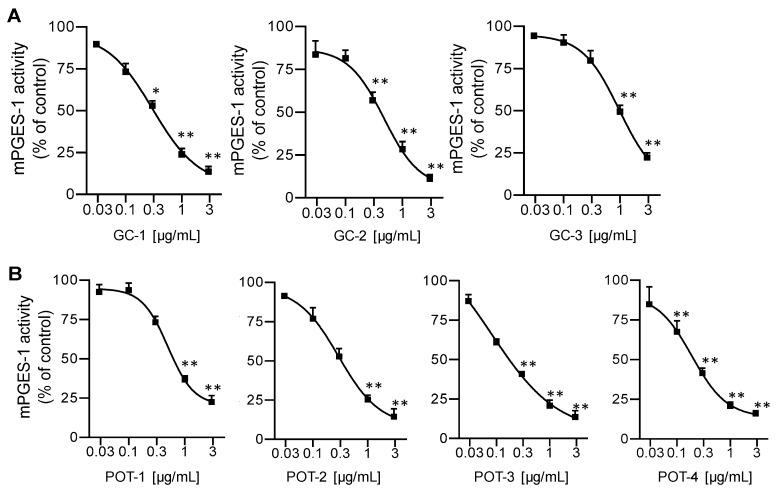
Inhibition of mPGES-1 in a cell-free assay by (**A**) extracts prepared from *G. cambogia* using *n*-hexane (GC-1), DCM (GC-2), or ethyl acetate (GC-3), and by (**B**) extracts of *P. scandens* using *n*-hexane (POT-1), DCM (POT-2), ethyl acetate (POT-3), or methanol (POT-4). Data are given as means +/− S.E.M, *n* = 3. * *p* < 0.05; ** *p* < 0.01.

A number of 5-LO inhibitors of natural origin are also capable of interfering with PGE_2_ formation by blocking mPGES-1 [8]. Hence, we evaluated the extracts also for potential mPGES-1 inhibitory activity in a cell-free assay. Again, extracts were first screened at 10 µg/mL, and for those that caused >50% inhibition, the IC_50_ values were determined. Among the seven plants, only extracts from *G. cambogia* and *P. scandens* were active against mPGES-1. As for inhibition of 5-LO, the lipophilic *n*-hexane, DCM and ethyl acetate extracts of *G. cambogia* displayed potent mPGES-1 inhibitory activities with IC_50_ values of 0.29, 0.49, 0.85 µg/mL, respectively (Figure 2A). Of interest, although 5-LO inhibition was not observed for extracts prepared from *P. scandens*, those preparations efficiently inhibited mPGES-1 with the ethyl acetate extract being most potent (IC_50_ = 0.10 µg/mL, Table 1, Figure 2B). Note that these active plant extracts possessed very low IC_50_ values, with superior potency over the reference mPGES-1 inhibitor MK886 (IC_50_ = 1.2 μg/mL, Table 1).

### 2.3. NO Scavenging Activity 

Since the expression of the inducible isoform of NOS, connected to massive NO production, has been proposed as an important component of inflammation [20,21], we evaluated the NO scavenging activity of the plant extracts in a cell-free assay according to Boora et al. (2014) [22]. Out of the tested plants, extracts of *H. fucatus, M. cerviana, N. arbor-tristis* and *O. mungos* displayed NO scavenging activity by ≥50% at an initial concentration of 1000 μg/mL. These extracts were further subjected to concentration-response experiments and the obtained IC_50_ values are shown in Table 2. In contrast to inhibition of 5-LO and mPGES-1, we observed NO scavenging activities also for extracts based on the polar solvent methanol. The reference drug aspirin displayed pronounced NO scavenging activity with an IC_50_ of 3.5 μg/mL, as expected.

### 2.4. Antibacterial Activity

Among the tested extracts, only the extracts prepared from *G. cambogia* displayed antibacterial activity by inhibiting the growth of Gram-positive bacteria without any activity against the other bacterial species tested. The MIC values of extracts prepared from *G. cambogia* against *S. aureus*, *S. saprophyticus*, *E. feacalis* and MRSA strains are given in Table 3; cefotaxime and gentamicin were used as reference drugs. 

### 2.5. Disinfectant Activity

The disinfectant activity was assessed after treating smooth and rough surfaces with different plant extracts; the disinfectant Lifebuoy^®^ soap (containing silver as active ingredient [23]) was used as the reference. As shown in Table 4, a prominent disinfectant efficiency was observed especially with the *n*-hexane extract of *G. cambogia* against all the tested microorganisms on both types of surfaces. Testing of these organisms on smooth and rough surfaces against Lysol^®^ (benzalkonium chloride 0.10%) and Teepol^™^ (sodium dodecylbenzene sulfonate 10%) as disinfectant reference agents further confirmed the suitability and validity of our findings with the plant extracts. 

Furthermore, the mean bacterial colony count of an untreated surface was compared with that of a treated surface with the plant extracts (using the disinfectant Lifebuoy^®^ soap as reference) applying one-way ANOVA test (Table A2). Overall, according to the one-way ANOVA test there was a statistically significant reduction in MRSA colony counts on both types of surfaces, and *S. aureus* colony counts only on the smooth surface. Post-hoc comparisons found that there was no significant difference in reducing *S. aureus* and MRSA colony counts between the treatment with the commercial disinfectant Lifebuoy^®^ soap and the plant extracts on both types of surfaces. 

### 2.6. Phytochemical Analysis of the Most Potent Extracts

**Figure 3:**
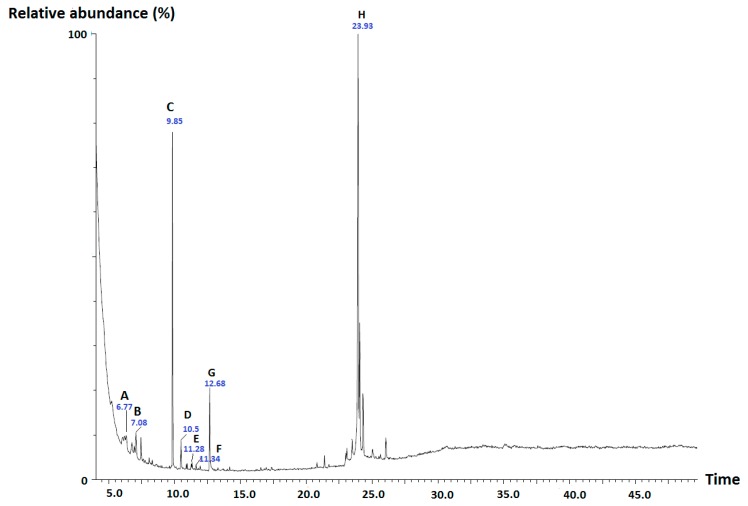
Total ion chromatogram of the *n*-hexane extract of *G. cambogia* and tentatively identified compounds. **A**: β-caryophyllene, **B**: α-caryophyllene, **C**: squalene-like compound with *m/z* 260, **D:** hexadecane, **E:** hexadecanoic acid, **F**: 5a-methoxy-9a-methyl-3,4,5a,6,7,8,9a,10-octahydropyrano[4,3-b]chromene-1,9-dione, **G**: 1-octadecanol, **H**: garcinol.

The promising results obtained with *n*-hexane and DCM extracts of *G. cambogia* in the mPGES-1 and 5-LO activity assays as well as in the antibacterial and disinfectant activity assays stimulated us to perform a GC-MS analysis of the extracts, which led to the identification of several components, based on the comparison of the obtained experimental mass spectra with those recorded in the NIST MS Search 2.0, and also by comparison with the respective standards. In both extracts, hexadecane, hexadecanoic acid, 5a-methoxy-9a-methyl-3,4,5a,6,7,8,9a,10-octahydropyrano[4,3-b]chromene-1,9-dione and 1-octadecanol were detected along with a prominent peak representing garcinol (based on comparison with the fragmentation pattern of the standard), a polyisoprenylated benzophenone derivative known to inhibit 5-LO and mPGES-1 [24] as well as another intense peak representing *m/z* value of 260 (Figure 3). Since there were numerous compounds suggested for *m/z* of 260 it was not possible to identify this peak. β-Caryophyllene and α-caryophyllene were exclusively present in the *n*-hexane extract while the presence of neophytadiene and α-tocopherol was observed only in the DCM extract.

**Figure A1:**
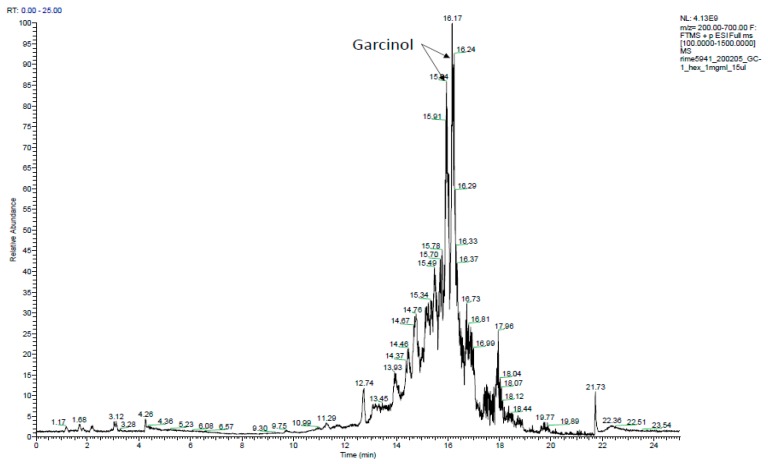

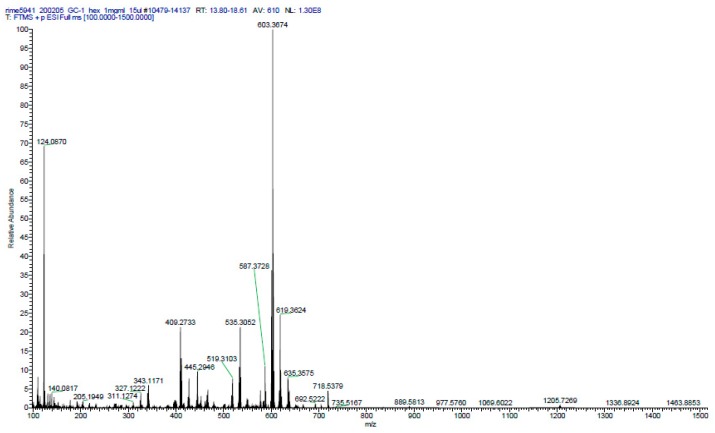
UPLC-MS analysis of the *n*-hexane extract of *G. cambogia* indicating the presence of garcinol.

**Figure A2:**
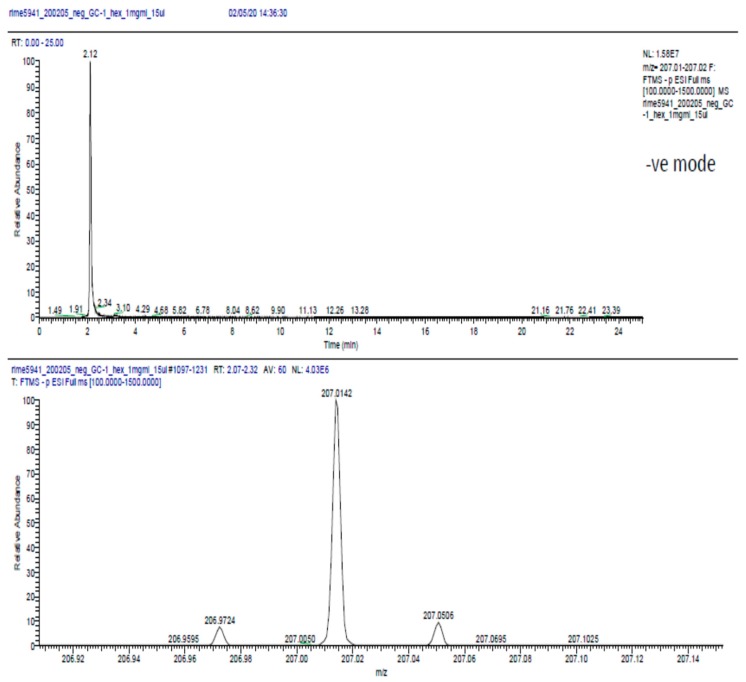
UPLC-MS analysis of the *n*-hexane extract of *G. cambogia* indicating the presence of hydroxycitric acid.

**Figure A3:**
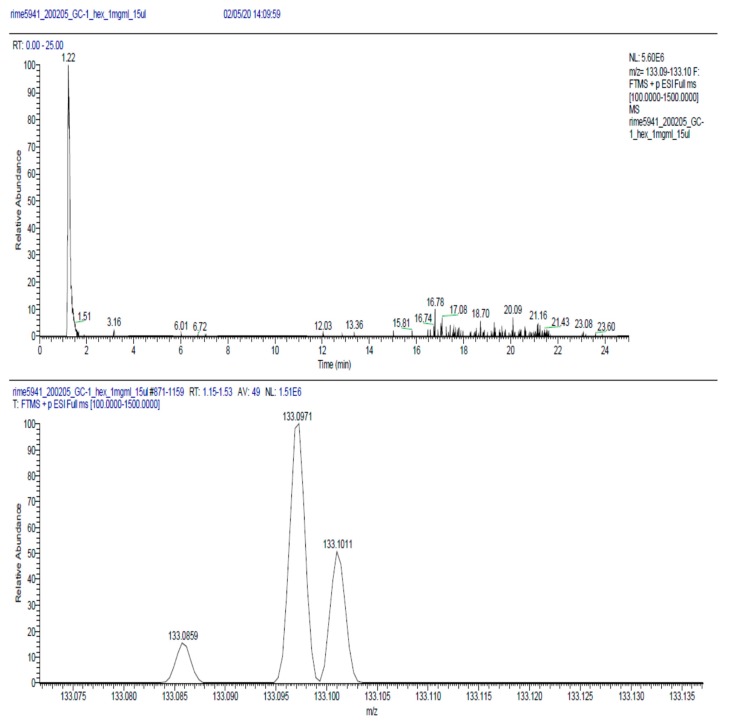
UPLC-MS analysis of the *n*-hexane extract of *G. cambogia* indicating the presence of ornithine.

**Figure A4:**
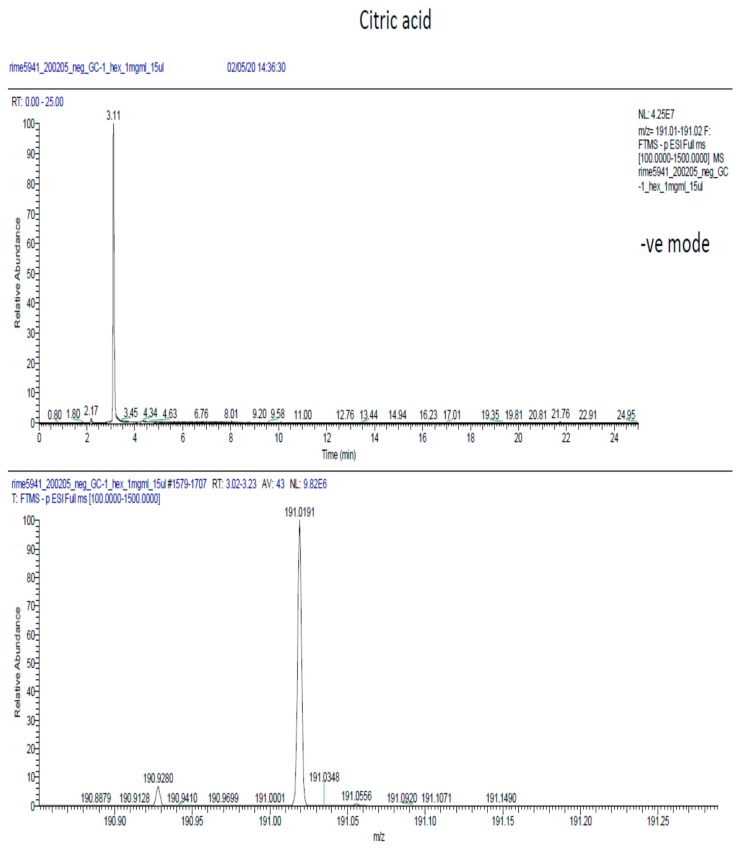
UPLC-MS analysis of the *n*-hexane extract of *G. cambogia* indicating the presence of citric acid.

**Figure A5:**
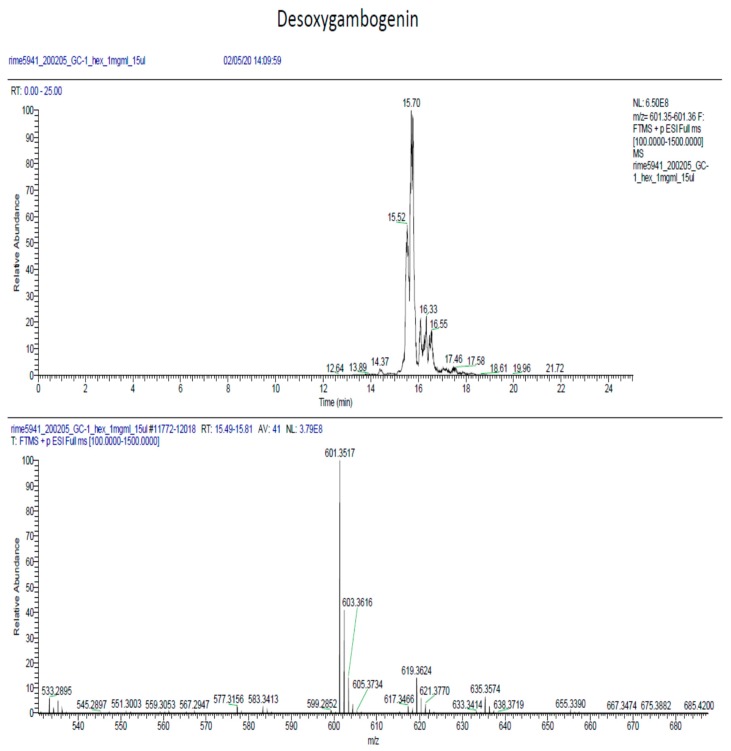
UPLC-MS analysis of the *n*-hexane extract of *G. cambogia* indicating the presence of desoxygambogenin.

**Figure A6:**
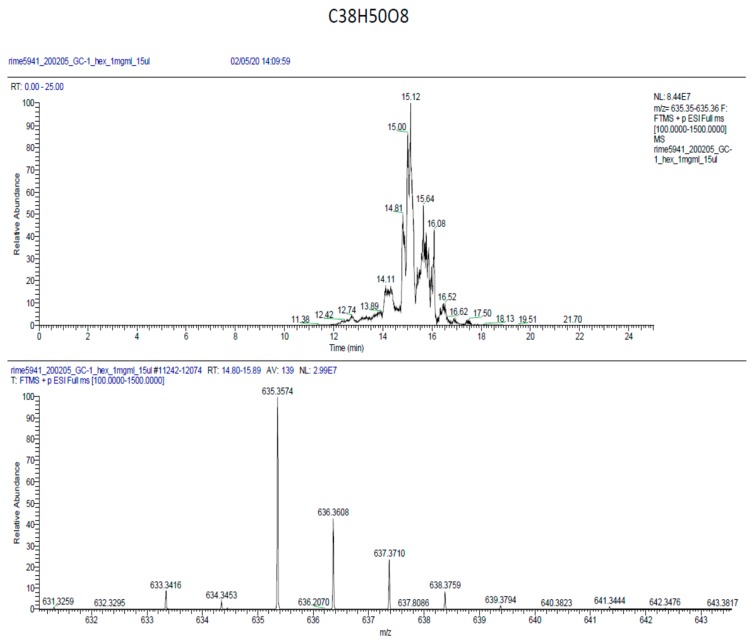
UPLC-MS analysis of the *n*-hexane extract of *G. cambogia* indicating the presence of a compound with a molecular formula of C_38_H_50_O_8_.

**Figure A7:**
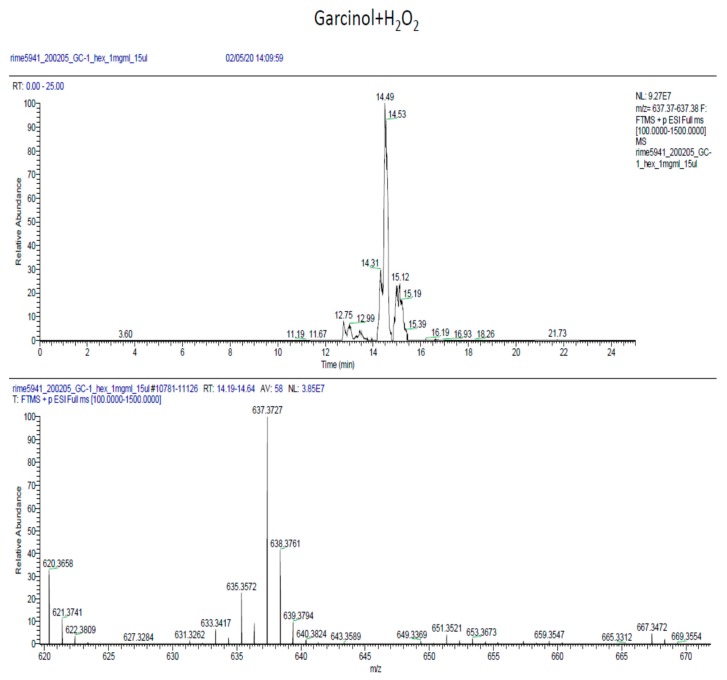
UPLC-MS analysis of the *n*-hexane extract of *G. cambogia* indicating the presence of the compound Garcinol+H_2_O_2_.

Moreover, using ultra-high performance liquid chromatography-tandem mass spectrometry (UPLC-MS/MS), the following compounds were identified based mass features: hydroxycitric acid [M + H]+ m/z 209.0290 (−0.831 ppm) C_6_H_9_O_8_; ornithine [M + H]^+^ *m*/*z* 133.0972 (0.494 ppm) C_5_H_13_N_2_O_2_; citric acid [M − H]^−^ *m*/*z* 191.0191 (−3.32 ppm) C_6_H_7_O_7_; garcinol [M + H]^+^ *m*/*z* 603.3671 −1.567 ppm) C_38_H_51_O_6_; desoxygambogenin (garcinol with an extra unsaturation) [M + H]^+^ *m*/*z* 601.3519 (−0.795 ppm) C_38_H_49_O_6_; [M + H]^+^ *m*/*z* 635.3573 (−0.795ppm) C_38_H_51_O_8_, and garcinol+H_2_O_2_ [M + H]^+^ *m*/*z* 637.3724 (−1.718 ppm) C_38_H_53_O_8_. Garcinol was identified as the major compound (Figure A1) in these extracts and TICs, and extracted ion chromatograms of the other identified compounds are given in Figure A2, Figure A3, Figure A4, Figure A5, Figure A6 and Figure A7. The UPLC-MS/MS-based phytochemical analysis of the methanolic extract of *O. mungos* that had displayed both 5-LO inhibition and NO scavenging activity was found to contain camptothecin, an alkaloid with anticancer activity [25] along with its glycoside derivatives.

## 3. Discussion

Traditional health care systems based on plants and plant-derived products are highly popular and employed therapeutically in Sri Lanka. Moreover, the medicinal plants utilized in these indigenous systems of medicine might be exploited as renewable sources for novel pharmacological agents. The present investigation was carried out to explore the anti-inflammatory and antimicrobial potential of several medicinal plants that are widely employed in folklore medicine in Sri Lanka. Since there is a dearth of in-depth studies that evaluated the molecular mechanisms underlying the anti-inflammatory activities of these Sri Lankan medicinal plants as well as the potential applications of herbal antimicrobials, our study provides new insights into the field of medicinal plant research in Sri Lanka.

In order to determine the anti-inflammatory features of the selected plant species, we have focused on their ability to control major pro-inflammatory mediators. Among the numerous pro-inflammatory molecules that are synthesized and secreted during inflammatory responses, eicosanoids are crucial signaling lipid mediators derived from the metabolism of arachidonic acid and include pro-inflammatory prostaglandins (particularly PGE_2_) and leukotrienes but also anti-inflammatory lipoxins [26]. Arachidonic acid is first liberated from membrane phospholipids by the action of phospholipase A_2_ (PLA_2_), and then cyclooxygenases (COX-1 and COX-2) convert free arachidonic acid into the unstable intermediate PGH_2_. Thereafter, PGE_2_ synthase (PGES) enzymes isomerize PGH_2_ into PGE_2_ [27]. Leukotrienes are produced from arachidonic acid by the initial action of the key enzyme 5-LO [28]. The suppression of the biosynthesis of eicosanoids is an important therapeutic approach in the management of inflammatory conditions. For example, aspirin and indomethacin belong to the first generation of NSAIDs that inhibit both COX-1 and COX-2 and evoke severe unwanted side effects, while celecoxib belongs to the second generation of NSAIDs that selectively inhibit COX-2 [29]. However, selective COX-2 inhibitors are associated with cardiovascular risk due to the inhibition of the synthesis of physiologically required PGs (i.e., PGI_2_) downstream of PGH_2_. In this respect, inhibition of the inducible mPGES-1 appears as promising and ideal pharmacological strategy that is capable of blocking only the production of pro-inflammatory PGE_2_ without affecting the formation of PGI_2_ and other PGs and thus, with lower risk of adverse reactions [30,31]. Moreover, dual interference with mPGES-1 and 5-LO might be more efficient to reduce inflammatory deregulation, and popular herbal remedies such as frankincense, cannabis, St. John’s wort and isolated natural products like curcumin, EGCG, garcinol, carnosol and carnosic acid have been recognized as dual inhibitors of the two enzymes [8,32].

Our investigations revealed that the 5-LO-inhibitory potential of *n*-hexane and DCM extracts of *G. cambogia* is outstanding (IC_50_ = 0.15–1.39 µg/mL) in comparison to other potent Sri Lankan plant extracts (IC_50_ = 0.45–12 µg/mL) that were previously reported by us [10,11,12] as well as to various other plant-derived extracts (IC_50_ = 15–50 µg/mL) [33], and is comparable to that of the reference drug zileuton (IC_50_ = 0.11–0.13 µg/mL). Furthermore, these *n*-hexane and DCM extracts efficiently inhibited mPGES-1 activity (IC_50_ = 0.29 and 0.49 µg/mL, respectively). Therefore, our data suggest a high pharmacological potential of the *G. cambogia* extracts as dual 5-LO/mPGES-1 inhibitors in comparison to other well-recognized natural products that dually block 5-LO and mPGES-1 [8]. Along these lines, anti-inflammatory activities of *G. cambogia* extracts in animal models were reported [34,35], where suppression of PGE_2_ and leukotrienes may eventually contribute. Interestingly, all extracts prepared from *P. scandens* are exceptionally potent as selective inhibitors of mPGES-1 (IC_50_ = 0.1–0.5 µg/mL), supporting the interference with mPGES-1 as underlying anti-inflammatory mechanism of *P. scandens*. Especially the mPGES-1 inhibition by the ethyl acetate extract of *P. scandens* appeared remarkable (IC_50_ = 0.1 µg/mL) as such a high potency has not been reported for any plant extract yet.

The inducible NOS is associated with inflammatory conditions, resulting in massive production of NO [20]. Recent investigations on the pharmacological suppression of NO formation have focused on the blockade of NOS, inhibition of downstream mediators, or NO inhibition/scavenging [36,37]. Extracts from a number of plants such as *Alstonia scholaris*, *Cynodon dactylon*, *Morinda citrifolia* [38], *Hypericum rumeliacum*, *H. richeri* and *H. tetrapterum* [39] demonstrated NO scavenging activity. However, the NO scavenging potencies of these extracts were lower than those found for plant extracts of the present study, e.g., the methanol extract of *O. mungos* and the DCM extract of *H. furcatus*. Note that the methanol extract of *O. mungos* is the only one among all 28 studied extracts that displayed 5-LO- or mPGES-1-inhibitory activity in addition to NO scavenging activity, suggesting distinct active principles in the various extracts for these targets.

In addition to the anti-inflammatory activity studies, the antibacterial potency of these plants was evaluated against both Gram-positive and Gram-negative bacterial species while the disinfectant properties were also determined. The Gram-positive organisms used in this study can switch from a commensal to a pathogenic status and are responsible for diseases of human skin and body surfaces. The rationale for using MRSA is that these strains are a leading cause of nosocomial infections and only a limited number of antibiotics are available as efficient treatment options. Although the investigated plant species are reputed as antimicrobial therapeutics, our investigations revealed that except for *G. cambogia*, the antibacterial potential is negligible in all the other plants and extracts thereof. The antibacterial activity of *G. cambogia* extracts was demonstrated before [35] and the lipophilic extracts of *G. cambogia* used in the present study showed pronounced effects in this respect against *S. aureus*, *S. saprophyticus* and four strains of MRSA (MIC = 31.25–125 µg/mL). These MIC values are lower than those reported for most of the ubiquitous phyto-constituents in the literature [40,41]. Moreover, the disinfectant potency of the *G. cambogia* extracts was comparable to that of the commercial disinfectant Lifebuoy^®^ soap used as reference [23]. Although the disinfectant capacity of an obicure tea extract containing green tea, *G. cambogia*, ginger and pepirene was determined from zone of inhibition as well as by totally incubating in a broth and visualizing the presence or absence of turbidity [42], to the best of our knowledge, this is the first report on the evaluation of disinfectant potency of *G. cambogia* extract on different surfaces that might correlate to smooth and rough surfaces in hospital settings. We have also tested Lysol^®^ (benzalkonium chloride 0.10%) and Teepol^™^ (sodium dodecylbenzene sulfonate 10%) using the same method to confirm our results with the *G. cambogia* extract. These standard disinfectants at the commonly used concentrations have shown 100% efficacy on eliminating the tested bacteria. However, benzalkonium chloride belongs to the cationic surfactants group of quaternary ammonium compounds that are found to be highly toxic to different aquatic organisms as well as being capable of inducing genotoxic effects in mammalian and plant cells [43]. Similarly, sodium dodecylbenzene sulfonate was reported to cause severe irritancy in rabbit skin at a concentration of 15% [44]. With the appearance and prevalence of multi-drug resistant microorganisms in the hospital settings along with the toxicity issues related to widely employed commercial disinfectants, the exploration of alternative antimicrobial agents from other sources, especially from plants is in high demand. In this regard, our findings may impose a significant impact towards the development of herbal disinfectants in a commercial scale.

In this study, we successively extracted the plant material with solvents of increasing polarity with the aim of relating the bioactivities to the chemical profile in the follow-up studies involved with activity-guided fractionation and compound identification. However, this could not be achieved with the extracts prepared with water or other solvents commonly used in traditional medicine. *n*-Hexane and DCM extracts of *G. cambogia* possessed most potent biological activities, and we thus attempted to get more insights into the identity of the respective bioactive agents by applying GC-MS or UPLC-MS/MS analysis. Among the identified compounds several have already been reported to exert anti-inflammatory activities, for example, the β-caryophyllene and α-caryophyllene [45,46] present in the *n*-hexane extract, and α-tocopherol [47] in the DCM extract. Of particular interest is garcinol, a potent dual inhibitor of 5-LO and mPGES-1 (IC_50_ = 0.1 and 0.3 µM), present in the fruit rind of Clusiaceae species [24], which we identified as the major constituent in the present extracts. Interestingly, the IC_50_ values (cell-free assay for 5-LO and mPGES-1) obtained for the *n*-hexane extract of *G. cambogia*; i.e., IC_50_ = 0.15 µg/mL for 5-LO and 0.29 µg/mL for mPGES-1 would correspond to 0.25 and 0.48 µM garcinol, respectively, being comparable to the reported IC_50_ values [24]. Furthermore, potent antimicrobial activity for garcinol isolated from *G. purpurea* against some MRSA strains with MIC = 6.25–25 µg/mL was shown [48] and these results would be compatible with the MIC value of 31.25 µg/mL of the *n*-hexane extract of G. *cambogia*. These observations suggest that garcinol significantly contributes to the anti-inflammatory and antibacterial activities of the extract. In addition, hydroxy citric acid, which is reported as a safe component [42] was also detected in the extracts of *G. cambogia*. Since the present study focused only on the dereplication of secondary metabolites in the bioactive extracts, further activity-guided fractionation is warranted for isolation and identification of the secondary metabolites in these bioactive extracts to confirm the role of specific secondary metabolites for the observed bioactivities.

## 4. Materials and Methods

### 4.1. Plant Material

Leaves of *H. furcatus*, *O. mungos*, *P. scandens* and whole plants of *A. populifolia*, along with flowers of *N. arbor-tristis* and fruits of *G. cambogia* were collected from home gardens in Nittambuwa, Gampaha District—Western Province of Sri Lanka in 2012 and 2015, while seeds of *M. cerviana* were purchased from Ayurvedic retail outlet at the Market Place, Nittambuwa, Sri Lanka. The selection of plant parts were based on their traditional utility. The plant materials were identified by the author M.N., a botanist, and confirmed based on the characteristics depicted in reference books [4,49]. The plant specimens were authenticated by comparison with the herbarium specimens at the National Herbarium, Royal Botanical Garden, Peradeniya, Sri Lanka and the voucher specimens of each plant were deposited at the Department of Biochemistry (Table A1), Faculty of Medicine, University of Ruhuna, Sri Lanka.

### 4.2. Preparation of Crude Extracts

Extraction of plant material was performed as described in our previous studies, where we evaluated the 5-LO- and mPGES-1-inhibitory effects of Sri Lankan plants, that is, *L. zeylanica* [10], *P. zeylanicus* [11] and *M. pinnata* [12]. In brief, the plant material was thoroughly washed with running water and dried in the shade (30 ± 2 °C) for five to seven days. Dried plant material was then powdered using a domestic grinder. Ten grams of each powdered material was successively extracted with 500 mL of *n*-hexane, dichloromethane (DCM), ethyl acetate, and methanol (Roth, Karlsruhe, Germany) at room temperature using a linear shaker for 50 min. The extracts were evaporated to dryness with the use of a rotary evaporator (BÜCHI, R-114, Essen, Germany), and solubilized in DMSO for bioactivity assays.

### 4.3. Evaluation of Bioactivities

#### 4.3.1. 5-LO Activity in Intact Neutrophils

Human neutrophils were isolated from leukocyte concentrates obtained from the University Hospital Jena, Germany. In brief, peripheral blood was withdrawn from fasted (12 h) healthy donors with consent that had not taken any anti-inflammatory drugs during the last 10 days, by venipuncture in heparinized tubes (16 IE heparin/mL blood). The approval for the protocol was given by the ethical committee of the University Hospital Jena and all methods were performed in accordance with the relevant guidelines and regulations. The blood was centrifuged at 4000× *g* for 20 min at 20 °C. Leukocyte concentrates were then subjected to dextran sedimentation and to centrifugation on Nycoprep cushions (PAA Laboratories, Linz, Austria). Contaminating erythrocytes of pelleted neutrophils were lysed by hypotonic lysis using water. Neutrophils were washed twice in ice-cold PBS and finally resuspended in PBS pH 7.4 containing 1 mg/mL glucose and 1 mM CaCl_2_ (PGC buffer) (purity > 96–97%). The cells were pre-incubated for 15 min at 37 °C with test compounds or vehicle (0.1% DMSO) and incubated for 10 min at 37 °C with Ca^2+^-ionophore A23187 (2.5 µM) plus 20 µM arachidonic acid. Then, the reaction was stopped on ice by addition of 1 mL of methanol, 30 µL 1 N HCl and 500 µL PBS, and 200 ng prostaglandin B_1_ were added. The samples were subjected to solid phase extraction on RP18-columns (100 mg, UCT, Bristol, PA, USA) and 5-LO products (LTB_4_ and its trans-isomers, 5-hydro(pero)xyeicosatetraenoic acid (H(P)ETE)) were analyzed by HPLC on the basis of the internal standard PGB_1_. Cysteinyl-LTs C_4_, D_4_ and E_4_ were not detected (amounts were below detection limit), and oxidation products of LTB_4_ were not determined. Zileuton was used as reference drug [18].

#### 4.3.2. 5-LO Activity in Cell-Free Assays

*E. coli* (BL21) were transformed with pT3-5-LO plasmid, and recombinant 5-LO protein was expressed and partially purified as described [50]. In brief, cells were lysed in 50 mM triethanolamine/HCl pH 8.0, 5 mM EDTA, soybean trypsin inhibitor (60 µg/mL), 1 mM phenyl-methanesulphonyl fluoride, and lysozyme (1 mg/mL), homogenized by sonication (3 × 15 s), and centrifuged at 40,000× *g* for 20 min at 4 °C. The 40,000× *g* supernatant was applied to an ATP-agarose column to partially purify 5-LO as described [44]. Aliquots of semi-purified 5-LO were immediately diluted with ice-cold PBS containing 1 mM EDTA, and 1 mM ATP was added. Samples were pre-incubated with the test compounds or vehicle (0.1% DMSO) as indicated. After 10 min at 4 °C, samples were pre-warmed for 30 s at 37 °C, and 2 mM CaCl_2_ plus 20 µM arachidonic acid was added to start 5-LO product formation. The reaction was stopped after 10 min at 37 °C by the addition of 1 mL ice-cold methanol, and the formed metabolites were analyzed by RP-HPLC as described [50]. 5-LO products include the all-trans isomers of LTB_4_ as well as 5-HPETE and its corresponding alcohol 5-HETE. Zileuton was used as reference drug.

#### 4.3.3. Determination of mPGES-1 Activity

Microsomal preparations of A549 cells were prepared as previously described [51]. Briefly, A549 cells were cultured in Dulbecco’s Modified Eagle’s Medium (DMEM) containing 2% FCS and IL-1β (2 ng/mL) for 72 hrs (37 °C, 5% CO_2_). Cells were then harvested and resuspended in homogenization buffer (potassium phosphate (0.1 M, pH 7.4), phenylmethanesulfonyl fluoride (1 mM), soybean trypsin inhibitor (60 µg/mL), leupeptin (1 µg/mL), glutathione (2.5 mM), and sucrose (250 mM)). After shock-freezing of the cells in liquid nitrogen, sonication (3 × 20 s), differential centrifugation at 10,000× *g* (10 min, 4 °C) and 174,000× *g* (60 min, 4 °C), the pellets were re-suspended in homogenization buffer. The microsomes were diluted in potassium phosphate buffer (0.1 M, pH 7.4) with glutathione (2 mM) and pre-incubated with the test compounds or vehicle (0.1% DMSO) on ice for 15 min. After stimulation (1 min, 4 °C) with 20 µM PGH_2_ as substrate, the reaction was terminated by addition of stop solution containing FeCl_3_ (40 mM), citric acid (80 mM), and 11β-PGE_2_ (10 µM as internal standard) and analyzed for PGE_2_ by RP-HPLC as reported before [51]. MK886 was used as reference drug.

#### 4.3.4. NO Scavenging Activity

The nitric oxide scavenging assay was performed following the method described by Boora et al. with slight modifications [22]. Sodium nitroprusside (10 mM) in PBS (0.5 mL) was mixed with 1 mL of the plant extracts (1000 µg/mL) and incubated at 25 °C for 180 min. The extract was mixed with an equal volume (1.5 mL) of freshly prepared Griess reagent consisting of 1% sulphanilamide in 2% phosphoric acid and 0.1% naphthylethylene diamine dihydrochloride in 2% phosphoric acid. The same procedure was followed for the control sample containing an equal volume of buffer instead of the plant extract. The absorbance was measured at 546 nm using a UV-visible spectrophotometer (UV_1800, Shimadzu, Kyoto, Japan). The percentage inhibitory activity by the plant samples was calculated using the following formula:

Inhibition (%) = {(A_control_ − A_test_)/A_control_} × 100 (1)

Based on the preliminary observations, extracts that displayed an inhibition ≥50% at 1000 µg/mL were further subjected to concentration-response studies. Aspirin was used as reference drug [52].

#### 4.3.5. Antibacterial Activity

Antibacterial activity was assessed using standard bacterial cultures, *Staphylococcus aureus* (ATCC 25923), *Enterococcus faecalis* (ATCC 29212) and *Escherichia coli* (ATCC 35218) obtained from the Department of Microbiology, Faculty of Medicine, University of Ruhuna, Sri Lanka. In addition, the antibacterial activity of the plant extracts was determined against clinical isolates including *Staphylococcus saprophyticus*, *Salmonella* Typhi and four strains of methicillin-resistant *Staphylococcus aureus* (MRSA) provided by the Department of Microbiology, Faculty of Medicine, University of Ruhuna, Sri Lanka.

A loop full from an isolated colony of one-day-old cultures was dissolved in sterile distilled water and the turbidity of the mixture was adjusted equally to that of the McFarland 0.5 standard. Broth microdilution method, described by Napagoda et al., 2018 [10], was employed with slight modification to determine the antimicrobial activity and the minimum inhibitory concentrations (MIC) of plant extracts. Briefly, 100 µL of solvent controls and test samples were added to the first wells of the microplate starting with a concentration of 2 mg/mL and were then two-fold serially diluted down the wells. 100 µL of the diluted culture with a turbidity standard equal to 0.5 McFarland and 50 µL of Muller Hinton Broth (MHB) was added to all wells. The microtiter plates were incubated for 24 h at 37 °C. After incubation, resazurin powder dissolved in phosphate buffered saline (pH 7.4) to a final concentration of 0.1 mg/mL was added to all wells (30 µL per well), and further incubated for 2 h for the observation of color change. The MIC was determined as the lowest concentration of test agent that prevents the growth of bacteria and was identified as the concentration which could not change the initial blue color of resazurin dye into pink. MBC (minimum bactericidal concentration) is the lowest concentration of an antibacterial agent required to kill a particular bacterium and was determined by sub-culturing the content of the above microtiter plate wells in agar plates. The assay was conducted in triplicates. Gentamicin and cefotaxime were used as reference drugs for the antimicrobial assay.

#### 4.3.6. Disinfectant Potency

The disinfectant potency was determined against *S. aureus* and against two clinical isolates of MRSA following the method described by Singh et al. (2012) [53], with slight modifications. Pre-autoclaved rough (floor tile) and smooth (stainless steel) surfaces (50 cm^2^) were treated evenly with 1 mL of the bacterial suspension (equal to McFarland 0.5 turbidity) and allowed to dry for 1 h. In each surface, two squares (25 cm^2^) were labeled and the test solution (2.5 mL at its MBC) was applied by a sterile cotton gauge in one square, while the other (labeled as non-disinfected area) was left without any treatment. After a contact period of 10 min, both areas were swabbed and each swab was vortexed in a tube containing 5 mL of MHB and a dilution was prepared as 1:10. Five drops of the dilution were inoculated on Muller Hinton Agar (MHA) plates and incubated for 48 h. The commercial disinfectant Lifebuoy^®^ soap (Unilever, London, UK) as well as Lysol^®^ (benzalkonium chloride 0.10%) and Teepol^™^ (sodium dodecylbenzene sulfonate 10%) were used as positive controls. The experiment was performed in duplicates. The disinfectant potency was calculated as:

Disinfectant potency = 100 − [number of colonies on treated plate/number of colonies on untreated plate] × 100(2)

The data were analyzed by Statistical Package for the Social Sciences (SPSS) version 15 (https://spss-for-windows-evaluation-version.updatestar.com/).

#### 4.3.7. Gas Chromatography Coupled to Mass Spectrometric Analysis

Out of the tested crude extracts, a remarkable bioactivity was observed in *n*-hexane and DCM extracts of *G. cambogia*, which were hence subjected to the phytochemical profiling following the method described by us before [10,11,12]. Briefly, the dried crude extract was dissolved in ethyl acetate (1 mg/mL) and analyzed on a gas chromatograph HP6890 (Agilent, Santa Clara, CA, USA) connected to a MS02 mass spectrometer from Micromass (Waters, Manchester, UK) with EI 70 eV using a ZB-5ms column (30 m × 0.25 mm, 0.25 μm film thickness; Phenomenex, Torrance, CA, USA). The carrier gas was helium at a flow rate of 1 mL/min. The injector temperature was kept at 250 °C and the temperature program was set as 100 °C (2 min), 15 °C/min to 200 °C, 5 °C/min to 305 °C (20 min).

#### 4.3.8. Ultra-High Performance Liquid Chromatography-Tandem Mass Spectrometry (UPLC-MS/MS)

In addition, analyses of the *G. cambogia* extracts were performed on a QExactive-HF-X and an Ultimate 3000 series RSLC (Dionex, Sunnyvale, CA, USA) chromatography system. Chromatographic separation was achieved on an Acclaim C_18_ column (150 × 2.1 mm, 2.2 µm particles with 120 Å pore diameter, Dionex) with a flow rate of 300 µL min^−1^ in a binary solvent system of water (Solvent A) and acetonitrile (Solvent B), both containing 0.1% (*v*/*v*) formic acid. 15 µL each of extract was loaded onto the column and eluted by using a gradient as follows: linear increase from 0% B to 100% B within 15 min–100% B constant for 5 min–equilibration time at 0% B for 5 min. The mass spectrometer was operated in positive and negative ionization modes using Heated-Electrospray Ionization (H-ESI). The source parameters were set to 4 kV for spray voltage, 35 V for transfer capillary voltage, capillary temperature 300 °C and Funnel RF of 40 V. Fragmentation was done using data-dependent acquisition mode with MS1 full scan at 100–1500 *m/z* at 60,000 m/Δm resolving power and up to five MS/MS scans (TOP5) of the most abundant ions per duty cycle with 30,000 m/Δm resolving power and stepped normalized collision energy of 20, 30 and 40. A mass tolerance of ± 4 ppm was used as threshold between accurate and exact mass. Data was evaluated and interpreted using Xcalibur v.3.0.63 software (Thermo Fisher Scientific, Waltham, MA, USA). In addition, UPLC-MS/MS analysis was also performed for the methanolic extract of *O. mungos* that had displayed both 5-LO inhibition and NO scavenging activity.

#### 4.3.9. Statistical Analysis

Data were expressed as mean ± S.E.M. The IC_50_ values were calculated from averaged measurements at 4–5 different concentrations of the compounds by nonlinear regression using GraphPad Prism software (GraphPad Software, San Diego, CA, USA) one site binding competition. Statistical evaluation of the data was performed by one-way ANOVA followed by a Bonferroni or Tukey-Kramer post-hoc test for multiple comparisons respectively. A *p* value < 0.05 was considered significant.

## 5. Conclusions

The present study provides important insights into the biological activities of several anti-inflammatory medicinal plants from Sri Lanka. Both 5-LO and mPGES-1 are key enzymes in the production of the major pro-inflammatory lipid mediators LTB_4_ and PGE_2_, respectively, and our data unequivocally show that the active extracts directly inhibit these enzymes. Drugs interfering with these lipid mediators (e.g., NSAIDs, zileuton, montelukast) are on the market to treat inflammation. Thus, the detection of highly potent 5-LO and/or mPGES-1 inhibitory activities, especially in *G. cambogia* and *P. scandens* extracts, could be considered as supportive evidence for the traditional claims of these medicinal plants as anti-inflammatory remedies. Certainly, additional modes of actions of these extracts (e.g., interference with cytokine/chemokine release, suppression of the expression of COX-2, iNOS, adhesion molecules etc.) may contribute to the overall anti-inflammatory properties as well. Characterization of secondary metabolites by GC-MS and UPLC-MS/MS analysis of the lipophilic extracts of *G. cambogia* revealed the presence of the dual 5-LO/mPGES-1 inhibitor garcinol that may be responsible for the high efficiency of the extracts in this respect. Although the antimicrobial potential in other plants is negligible, the findings for the lipophilic extracts of *G. cambogia* are highly promising and could direct towards the development of herbal disinfectants to combat nosocomial infections.

## Figures and Tables

**Table 1:** IC_50_ values of the investigated plant extracts for interference with 5-LO (cell-free and cell-based) and mPGES-1 (cell-free). MK886 and zileuton were used as reference drugs against mPGES-1 and 5-LO, respectively. For extracts that caused less than 50% inhibition at 10 µg/mL, IC_50_ values were not determined and are indicated as “-”. Data are given as means, *n* = 3.

Plant and Plant Extracts	IC_50_ Values (μg/mL)		
	5-LO (Cell-Free)	5-LO (Cell-Based)	mPGES-1 (Cell-Free)
***A. populifolia***			
*n*-Hexane	-	-	-
DCM	-	-	-
Ethyl acetate	-	-	-
Methanol	-	-	-
***G. cambogia***			
*n*-Hexane	0.15	0.92	0.29
DCM	0.16	1.39	0.49
Ethyl acetate	0.24	1.55	0.85
Methanol	-	-	-
***H. furcatus***			
*n*-Hexane	-	-	-
DCM	-	-	-
Ethyl acetate	1.0	10.0	-
Methanol	-	-	-
***M. cerviana***			
*n*-Hexane	5.5	6.8	-
DCM	-	-	-
Ethyl acetate	-	-	-
Methanol	-	-	-
***N. arbor-tristis***			
*n*-Hexane	-	-	-
DCM	-	-	-
Ethyl acetate	-	-	-
Methanol	-	-	-
***O. mungos***			-
*n*-Hexane	-	-	-
DCM	-	-	-
Ethyl acetate	-	-	-
Methanol	1.6	8.7	-
***P. scandens***			
*n*-Hexane	-	-	0.50
DCM	-	-	0.27
Ethyl acetate	-	-	0.10
Methanol	-	-	0.20
MK886	-	-	1.2
Zileuton	0.11	0.13	-

**Table 2:** IC_50_ values for the NO scavenging activity. Data are given as means, *n* = 3.

Plant Extract	IC_50_ (μg/mL)
*H. furcatus*-DCM	142.9
*H. furcatus*-methanol	229.4
*M. cerviana*-DCM	347.7
*M. cerviana*-ethyl acetate	760
*M. cerviana*-methanol	231.7
*N. arbor-tristis*-methanol	171.2
*O. mungos*–methanol	85.8
Aspirin (reference drug)	3.5

**Table 3:** MIC values of the different extracts prepared from *G. cambogia* against *S. aureus*, *S. saprophyticus*, *E. feacalis* and MRSA strains. Data are given as means, *n* = 3.

Extract/Drug	MIC (μg/mL)						
	*S. aureus*	*S. saprophyticus*	*E. feacalis*	MRSA 1	MRSA 2	MRSA 3	MRSA 4
*n*-Hexane	31.25	31.25	250	31.25	62.5	125	125
DCM	62.5	62.5	250	62.5	125	125	125
Ethyl acetate	500	500	-	-	-	-	-
Methanol	500	500	-	-	-	-	-
Cefotaxime	0.93	3.72	3.72	31.25	62.5	62.5	31.25
Gentamicin	7.5	7.5	31.25	31.25	31.25	31.25	15.6

**Table 4:** Disinfectant potency of *n*-hexane and DCM extracts prepared from *G. cambogia* against *S. aureus* and MRSA strains. Data are given as means, *n* = 3.

Tested Sample	Disinfectant Efficiency (%) against Different Microorganisms on Various Surfaces					
	*S. aureus*		MRSA Strain 1		MRSA Strain 2	
	Smooth Surface	Rough Surface	Smooth Surface	Rough Surface	Smooth Surface	Rough Surface
*n*-Hexane extract	100	100	87.5	100	80	78.5
DCM extract	58.3	60	90.6	100	69.2	100
Lifebuoy^®^ soap	86.3	40	92	55.5	100	20
Lysol^®^ (benzalkonium Chloride 0.10%)	100	100	100	100	100	100
Teepol^™^ (sodium dodecylbenzene sulfonate 10%)	100	100	100	100	100	100

## Abbreviations

COX	Cyclooxygenase
DCM	Dichloromethane
H(P)ETE	5-Hydro(pero)xyeicosatetraenoic acid
iNOS	Inducible nitric oxide synthase
LT	Leukotriene
5-LO	5-Lipoxygenase
mPGES-1	Microsomal prostaglandin E2 synthase-1
MHA	Muller Hinton Agar
MHB	Muller Hinton Broth
NO	Nitric oxide
NOS	Nitric oxide synthase
NSAID	Nonsteroidal anti-inflammatory drugs
PG	Prostaglandin
PLA_2_	Phospholipase A_2_
UPLC-MS/MS	Ultra-high performance liquid chromatography-tandem mass spectrometry

## Appendix

**Table A1:** The traditional uses of the plant species investigated in the present study.

Plant	Vernacular Name (in Sinhala)	Traditional Usage [4]	Voucher Specimen Number
*Argyreia populifolia*	Girithilla	Antiseptic, for rabid dog bites, to treat weak and spongy gum	MN_12_008
*Garcinia cambogia*	Goraka	Antiseptic, to treat skin infections, rheumatism, hypertension	MN_15_013
*Hibiscus furcatus*	Nabirtitha	Suppuration of boils, eye disease, as a remedy for swellings, for cleansing kidneys	MN_12_006
*Mollugo cerviana*	Pathpadagum	Antiseptic, cure itch and other skin diseases, to treat gonorrhoea, fever, cough and to reduce body odour	MN_12_005
*Nyctanthes arbor-tristis*	Sepalika	Carminative, stomachic, purgative, astringent, antibilious, expectorant, diuretic, treatment for piles, various skin diseases, fever, rheumatism	MN_15_009
*Ophiorrhiza mungos*	Dathkatiya	To treat snake bites, dressing ulcers and external tumors	MN_12_007
*Pothos scandens*	Potawel	cholagogues, diuretic, to treat wounds, rheumatic fever, chronic malarial fever, small pox, asthma, snake bites	MN_15_010

**Table A2:** Comparison of mean colony counts of different microorganisms on different surfaces after treatment with *G. cambogia* extracts by one-way ANOVA with post hoc multiple comparisons.

Test	Comparison of Mean Colony Counts of Different Microorganisms on Different Surfaces							
	*S. aureus*				MRSA			
ANOVA Test Result	Smooth Surface *p* = 0.01975		Rough Surface *p* = 0.07249		Smooth Surface *p* = 0.000038		Rough Surface *p* = 0.044589	
	Mean Colony Counts	Post Hoc Test vs. Untreated	Mean Colony Counts	Post Hoc Test vs. Untreated	Mean Colony Counts	Post Hoc Test vs. Untreated	Mean Colony Counts	Post Hoc Test vs. Untreated
Untreated surface	9.3		4.0		12.2		5.0	
*n*-Hexane extract	0.0	*p* = 0.0063	0.0	*p* = 0.0201	1.75	*p* = 0.000074	0.75	*p* = 0.0272
DCM extract	2.5	*p* = 0.0214	1.0	*p* = 0.0531	1.75	*p* = 0.000074	0.0	*p* = 0.0116
Lifebuoy^®^ soap	1.5	*p* = 0.0128	1.5	*p* = 0.0897	0.5	*p* = 0.000024	2.5	*p* = 0.1688

Comparison of mean colony counts of different microorganisms on different surfaces after treatment with *G. cambogia* extracts by one-way ANOVA with post hoc multiple comparisons.

Note—Testing of these organisms on two types of surfaces against Lysol^®^ (benzalkonium chloride 0.10%) and Teepol^™^ (sodium dodecylbenzene sulfonate 10%) revealed that they were able to completely eliminate these organisms.
